# Minimally invasive surgery versus laparotomy of nonmetastatic pT4a colorectal cancer: a propensity score analysis

**DOI:** 10.1097/JS9.0000000000000627

**Published:** 2023-07-18

**Authors:** Hui-Long Guo, Jing-Yao Chen, You-Zhen Tang, Qian-Lin Zeng, Qing-Long Jian, Ming-Zhe Li, Yu-Long He, Wen-Hui Wu

**Affiliations:** a Digestive Diseases Center; b Guangdong Provincial Key Laboratory of Digestive Cancer Research, The Seventh Affiliated Hospital of Sun Yat-sen University, Shenzhen; c Gastrointestinal Surgery Center, The First Affiliated Hospital of Sun Yat-sen University, Guangzhou, Guangdong Province, People’s Republic of China

**Keywords:** colorectal cancer, laparotomy, minimally invasive surgery, nonmetastatic pT4a colorectal cancer, propensity score matching

## Abstract

**Aim:**

The aim was to compare short-term and long-term oncological outcomes between minimally invasive surgery (MIS group) and laparotomy (lap group) in nonmetastatic pT4a colorectal cancer (CRC).

**Materials and methods:**

The study retrospectively analyzed the outcomes of 634 patients treated with radical operation from January 2015 to December 2021 for nonmetastatic pT4a CRC, with propensity score matching.

**Results:**

The conversion rate from the MIS group to laparotomy is 3.5%. Intraoperative blood loss, time to first anal exhaust, defecation and drainage tube removal, and complication rate were significantly less in the MIS group. After 5 years, the outcomes of the MIS group were no inferior to laparotomy outcomes [overall survival (OS): 72.7 vs. 77.8%, *P*=0.285; disease-free survival (DFS): 72.2 vs. 75.0%, *P*=0.599]. And multivariate analysis showed that age greater than or equal to 60 years old, lymph node metastasis and the carcinoembryonic antigen levels were independent variables for OS, while lymph node metastasis and CA125 levels were independent variables for DFS. The results of the graph show the relationship between the sum of scores of sex, age, complications, BMI, carcinoembryonic antigen, age, CA125, tumor site, N stage and tumor length diameter and 1-year, 3-year, and 5-year mortality and DFS of patients. Among them, tumor length diameter and N stage are significantly correlated with long-term survival and disease-free of patients.

**Conclusion:**

MIS is safe and feasible for nonmetastatic pT4a CRC, with the added benefit of accelerated postoperative recovery. In oncology, MIS did not affect OS and DFS.

## Introduction

Highlights

- The sample size is larger than in previous studies.
- The research method of propensity matching score is used to reduce the impact of confounding factors, which makes the results more reliable.
- R language was used to analyze the sum of scores of various categorical variables and continuous variables to predict 1-year, 3-year, and 5-year survival rates and tumor recurrence and/or metastasis rates of patients.

Colorectal cancer (CRC) is the third most common cancer around the world and second in terms of cancer-related mortality^1^. According to the Global Cancer Report 2020, about 3 002 899 people in China dies of cancer every year, and CRC was the second most common tumor in the country^2^. Surgical treatment is still the most impactful treatment for CRC^3^. The definition of pT4a CRC is tumor tissue invading the serosal surface (visceral peritoneum) or tumor perforation continuous with the serosal surface through inflammation^4^. And, nonmetastatic was defined as the absence of distant metastases but included lymph node metastases. For the past few years, with the development of minimally invasive surgery (MIS) and the advocation of the concept of enhanced recovery after surgery (ERAS), MIS was often mentioned in the surgical treatment of CRC, with its safety and reliability supported by a lot of studies^5–12^. Although there were a number of researches on pT4a CRC surgery, it remains controversial^13–16^.

The aim of this study was to analyze the short-term and long-term oncological outcomes in selected patients with nonmetastasis pT4a CRC through propensity score matching (PSM) analysis.

## Materials and methods

### Patients

The study has been approved by the institutional review board, and it abides by the revised Declaration of Helsinki and the requirements of good clinical practice. The work has been reported in line with the strengthening the reporting of cohort, cross-sectional and case–control studies in surgery (STROCSS) criteria^17^.

We retrospectively evaluated patients who underwent curative resection for histologically diagnosed nonmetastatic pT4a CRC from January 2015 to December 2021. The inclusion criteria are as follows: pathologically diagnosed nonmetastatic pT4a CRC; underwent radical surgery; complete follow-up data were available, no contraindications before operation. Patients with metastasis, treated with nonradical surgery and a history of other tumors were excluded. Right colon cancer included ascending colon cancer and cancer of the hepatic flexure of the colon, and left colorectal cancer included cancer of the splenic flexure of the colon, cancer of the descending colon, sigmoid colon cancer, and rectal cancer. In addition, well differentiated means high differentiation, medium-high differentiation and medium-low differentiation, and poorly differentiated means medium-low differentiation, low differentiated and undifferentiation. Individual consent for this retrospective analysis was waived.

### Surgical techniques

All patients were stratified into two groups according to their surgical approach. All operations were performed by experienced gastrointestinal surgeons, and laparoscopy and robotic surgery are defined as MIS. The surgeries adopted radical principles, differing based on the operative site. Conversion was included in the group of Laparotomy when laparoscopy or robotic surgery found it difficult to achieve *en bloc* resection, complete hemostasis, or other unexpected situation.

### Follow-up

Other information collected included age at diagnosis, sex, BMI, postoperative hospital staying (days), intraoperative blood loss (ml), operative time (min), drainage tube removal time (days), antibiotic usage time (days), postoperative anal exhaust time (days), postoperative defecation time (day), neoadjuvant chemotherapy, pN stages, longitudinal diameter of tumor (cm), carcinoembryonic antigen (CEA), CA125, and CA199 levels, complications, and recurrence or metastasis. Abdominal computed tomography and tumor marker were monitored per 1–2 months after surgery and periodically after that. The median follow-up time was 46 months.

### Statistical analysis

Propensity scores were estimated using PSM with IBM SPSS statistics 25.0 software. The predictors included age, sex, BMI, tumor location, tumor differentiation level, pN stages, lymph node metastasis, and tumor longitudinal diameter. These parameters were chosen due to their significance while selecting surgical methods. The surgical approach was entered into the group indicator, and the match tolerance was 0.001. The name for the propensity variable was PM, and the case ID was the number of each surgical patient. The match ID variable name was E_ID, and the output dataset name was analyzed. In the end, data points with E_ID greater than or equal to 1 were chosen for further study.

All statistical analyses were performed with IBM SPSS Statistics 25.0 software. To evaluate differences in clinicopathological characteristics and short-term and long-term clinical outcomes among the groups, the *t*-test was used. Long-term outcomes and survival curves were analyzed using the Kaplan–Meier method. The logistic regression was used for multivariate analyses to identify independent prognostic factors for overall survival (OS) and relapse-free survival. Values of *P* less than 0.05 were regarded as statistically significant.

R language was used to draw a column chart, and categorical variables such as sex, complications, tumor site [left hemicolon carcer (LCC), right hemicolon carcer (RCC) and rectum carcer (ReC)] and continuous variables such as age, BMI, CEA, CA125, N stage, and tumor length and diameter were included in the score. The sum of the scores predicted 1-year, 3-year, and 5-year survival rates and the rate of tumor recurrence and/or metastasis.

## Result

### Prematch study population

One thousand three hundred and sixty-nine patients with nonmetastasis pT4a CRC who underwent curative resection from 2015 to 2021 were selected for this study. Tables 1 and 2 provided a good overview of the baseline characteristics of the prematched groups. There were multiple sets of significant imbalances.

**Table 1:** Clinicopathological characteristics of patients with nonmetastatic pT4a CRC.

	All (%)	Laparotomy (%)	Minimally invasive surgery (%)	*P*
*n*	1369	374 (27.3)	995 (72.7)	
Sex				0.570
Male	841 (61.4)	232 (16.9)	609 (44.5)	
Female	528 (38.6)	142 (10.4)	386 (28.2)	
Age (years)				<0.001
<60	586 (42.8)	140 (10.2)	446 (32.6)	
≥60	783 (57.2)	234 (17.1)	549 (40.1)	
Tumor location				<0.001
LCC	979 (71.5)	231 (16.9)	748 (54.6)	
RCC	390 (28.5)	143 (10.4)	247 (18.0)	
Differentiation				0.289
Well	1000 (73.0)	360 (14.4)	803 (58.6)	
Poor	369 (27.0)	72 (5.3)	297 (21.7)	
pN stages				0.055
N0	681 (49.7)	218 (15.9)	463 (33.8)	
NI	472 (34.5)	113 (8.3)	359 (26.2)	
N2	216 (15.8)	43 (3.1)	173 (12.6)	
Lymph node metastasis				<0.001
Yes	688 (50.3)	156 (11.4)	532 (38.9)	
No	681 (49.7)	218 (15.9)	463 (33.8)	
Tumor diameter longitudinal (cm)				<0.001
≥5 cm	500 (36.5)	153 (11.2)	347 (25.3)	
<5 cm	896 (63.5)	221 (16.1)	648 (47.3)	
CEA (ng/ml) >5 ng/ml				0.479
Yes	552 (40.3)	148 (10.8)	404 (29.5)	
No	817 (59.7)	226 (16.5)	591 (43.2)	
CA125 (ng/ml) >40 ng/ml				<0.001
Yes	87 (6.4)	42 (3.1)	45 (3.3)	
No	1287 (93.6)	332 (24.3)	950 (69.4)	
Conversion to laparotomy				
Yes			30 (3.0)	
No			965 (97.0)	
Complications (rate)		13.9	10.5	<0.001
Yes	152 (11.1)	52 (3.8)	100 (7.3)	
No	1217 (88.9)	322 (23.5)	995 (65.4)	

**Table 2:** Clinicopathological characteristics of patients with nonmetastatic pT4a CRC.

		All (%)	Laparotomy (%)	Minimally invasive surgery (%)	*P*
BMI	[median (IQR)]	22.0 (4.0)	22.0 (5.0)	22.0 (4.0)	0.013
Tumor diameter longitudinal (cm)	[median (IQR)]	4.0 (2.0)	4.0 (2.0)	4.0 (2.0)	0.039
Number of lymph nodes removed	[median (IQR)]	19.0 (15.0)	21.0 (16.0)	19.0 (14.0)	0.062
CEA (ng/ml)	[median (IQR)]	3.5 (7.3)	3.5 (8.2)	3.5 (7.2)	0.932
CA125 (ng/ml)	[median (IQR)]	10.1 (8.6)	10.7 (12.2)	9.9 (7.8)	0.119
Intraoperative blood loss (ml)	[median (IQR)]	50.0 (70.0)	100.0 (50.0)	50.0 (70.0)	<0.001
Operative time (min)	[median (IQR)]	214.0 (100.0)	195.0 (90.0)	220.0 (100.0)	<0.001
First exhaust (days)	[median (IQR)]	2.0 (2.0)	3.0 (2.0)	2.0 (2.0)	<0.001
First defecation (days)	[median (IQR)]	3.0 (2.0)	3.0 (3.0)	3.0 (2.0)	<0.001
Removal drainage tube (days)	[median (IQR)]	5.0 (3.0)	6.0 (2.0)	5.0 (3.0)	<0.001
Postoperative hospital staying (days)	[median (IQR)]	6.0 (3.0)	7.0 (4.0)	6.0 (3.0)	<0.001

### Postmatch baseline characteristics

**Figure 1:**
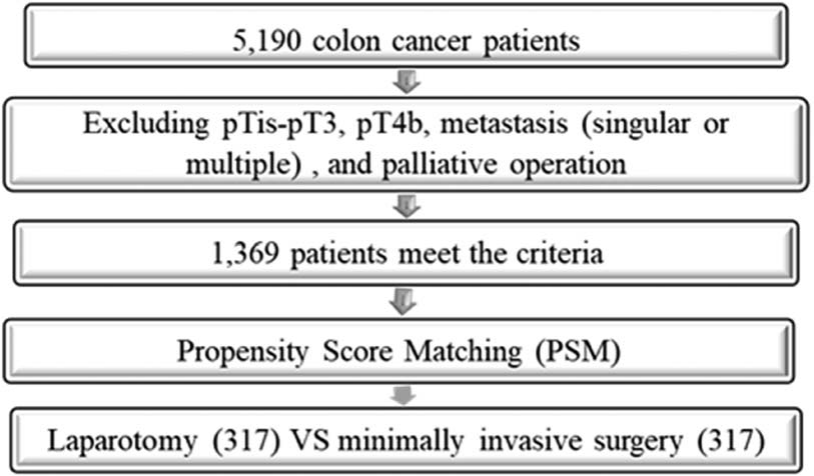
Flow chart of patient selection and propensity matching score matching process.

Details of the PSM process are shown in Figure 1. After matching, the statistical, and clinical features of all 634 of these matched patients are displayed in Tables 3 and 4. Eleven patients (3.5%) in the MIS group were converted to laparotomy. There were insignificant differences in sex, age, BMI, tumor longitudinal diameter, differentiation level, pN stages, lymph node metastasis, CEA, and CA125.

**Table 3:** Clinicopathological characteristics of patients with nonmetastatic pT4a CRC.

	All (%)	Laparotomy (%)	Minimally invasive surgery (%)	*P*
*n*	634	317 (50)	317 (50)	
Sex				0.514
Male	392 (61.8)	194 (30.6)	198 (31.2)	
Female	242 (38.2)	123 (19.4)	119 (18.8)	
Age (years)				0.105
<60	240 (37.9)	125 (19.7)	115 (18.1)	
≥60	394 (62.1)	192 (30.3)	202 (31.9)	
Tumor location				1.000
LCC	428 (67.5)	214 (33.8)	214 (33.8)	
RCC	206 (32.5)	103 (16.2)	103 (16.2)	
Differentiation				1.000
Well	516 (81.4)	258 (40.7)	258 (40.7)	
Poor	118 (18.6)	59 (9.3)	59 (9.3)	
pN stages				0.383
N0	346 (54.6)	171 (27.0)	175 (27.6)	
NI	202 (31.9)	106 (16.7)	96 (15.1)	
N2	86 (13.6)	40 (6.3)	46 (7.3)	
Lymph node metastasis				0.528
Yes	288 (45.4)	146 (23.0)	142 (22.4)	
No	346 (54.6)	171 (27.0)	175 (27.6)	
Tumor diameter longitudinal (cm)				
≥5 cm	248 (39.1)	122 (19.2)	126 (19.9)	0.516
<5 cm	386 (60.9)	195 (30.8)	191 (30.1)	
CEA (ng/ml) >5 ng/ml				0.516
Yes	248 (39.1)	122 (19.2)	126 (19.9)	
No	386 (60.9)	195 (30.8)	191 (30.1)	
CA125 (ng/ml) >40 ng/ml				0.695
Yes	27 (4.3)	13 (2.1)	14 (2.2)	
No	607 (95.7)	304 (47.9)	303 (47.8)	
Conversion to laparotomy				
Yes			11 (3.5)	
No			306 (96.5)	
Complications (rate)		15.2	11.7	0.010
Yes	85 (13.4)	48 (7.6)	37 (5.9)	
No	547 (86.6)	268 (42.4)	279 (44.1)	

**Table 4:** Clinicopathological characteristics of patients with nonmetastatic pT4a CRC.

		All (%)	Laparotomy (%)	Minimally invasive surgery (%)	*P*
BMI	[median (IQR)]	22.0 (4.0)	22.0 (4.0)	22.0 (4.0)	0.141
Tumor diameter longitudinal (cm)	[median (IQR)]	4.0 (1.5)	4.0 (2.0)	4.0 (1.0)	0.321
Number of lymph nodes removed	[median (IQR)]	20.0 (15.0)	20.0 (16.0)	19.0 (15.0)	0.527
CEA (ng/ml)	[median (IQR)]	3.4 (6.9)	3.5 (7.2)	3.4 (5.6)	0.942
CA125 (ng/ml)	[median (IQR)]	10.1 (8.6)	9.9 (9.4)	10.2 (8.1)	0.533
Intraoperative blood loss (ml)	[median (IQR)]	55.0 (50.0)	100.0 (50.0)	50.0 (60.0)	<0.001
Operative time (min)	[median (IQR)]	210.0 (100.0)	200.0 (95.0)	220.0 (95.0)	<0.001
First exhaust (days)	[median (IQR)]	2.0 (2.0)	3.0 (2.0)	2.0 (2.0)	<0.001
First defecation (days)	[median (IQR)]	3.0 (2.0)	3.0 (3.0)	3.0 (2.0)	0.006
Removal drainage tube (days)	[median (IQR)]	5.0 (3.0)	6.0 (2.0)	5.0 (2.0)	<0.001
Postoperative hospital staying (days)	[median (IQR)]	7.0 (4.0)	7.0 (4.0)	6.0 (3.0)	<0.001

*Operative and postoperative outcomes*

**Figure 2:**
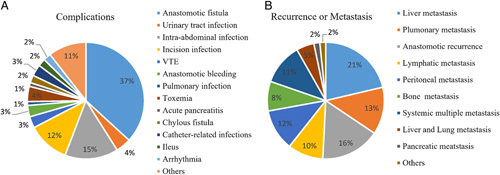
(A) In all patients who developed complications, the top three were anastomotic fistula (37%), intra-abdominal infection (15%), and incision infection (12%). (B) In patients with recurrence or metastasis, the top three were liver metastases (21%) and anastomotic recurrence.

Short-term outcomes are shown in Tables 3 and 4. The time of operation did significantly interfere with the procedure (laparotomy: 200.0±95.0 min vs. MIS: 220.0±95.0 min; *P*<0.001). Intraoperative blood loss in the MIS group (50.0±60.0 ml) was significantly less than that in the laparotomy group (100.0±50.0  ml; *P*<0.001). The first anal exhaust time was shorter in the MIS group (2.0±2.0 days) than the laparotomy group (3.0±2.0 days; *P*<0.001) and the first defecation time was significantly lower in the MIS group (3.0±2.0 days) than the laparotomy group (3.0±3.0 days; *P*=0.006). Moreover, the drainage tube removal time was significantly less in the MIS group (5.0±2.0 days) than the laparotomy group (6.0±2.0 days; *P*<0.001), and the postoperative hospital stay time was significant (MIS group: 6.0±3.0 days vs. 7.0±4.0 days; *P*<0.001). Finally, the rate of complication was significantly less in the MIS group (11.7%) than in the laparotomy group (15.2%; *P*=0.010). The incidence of complications is well reflected in Figure 2A (percentage of total complications). The most common one was anastomotic fistula (37%), followed by intra-abdominal infection (15%), incisional infection (12%), urinary tract infection (11%), VTE (3%), anastomotic bleeding (3%), pulmonary infection (3%), and others.

*Long-term outcomes*

**Figure 3:**
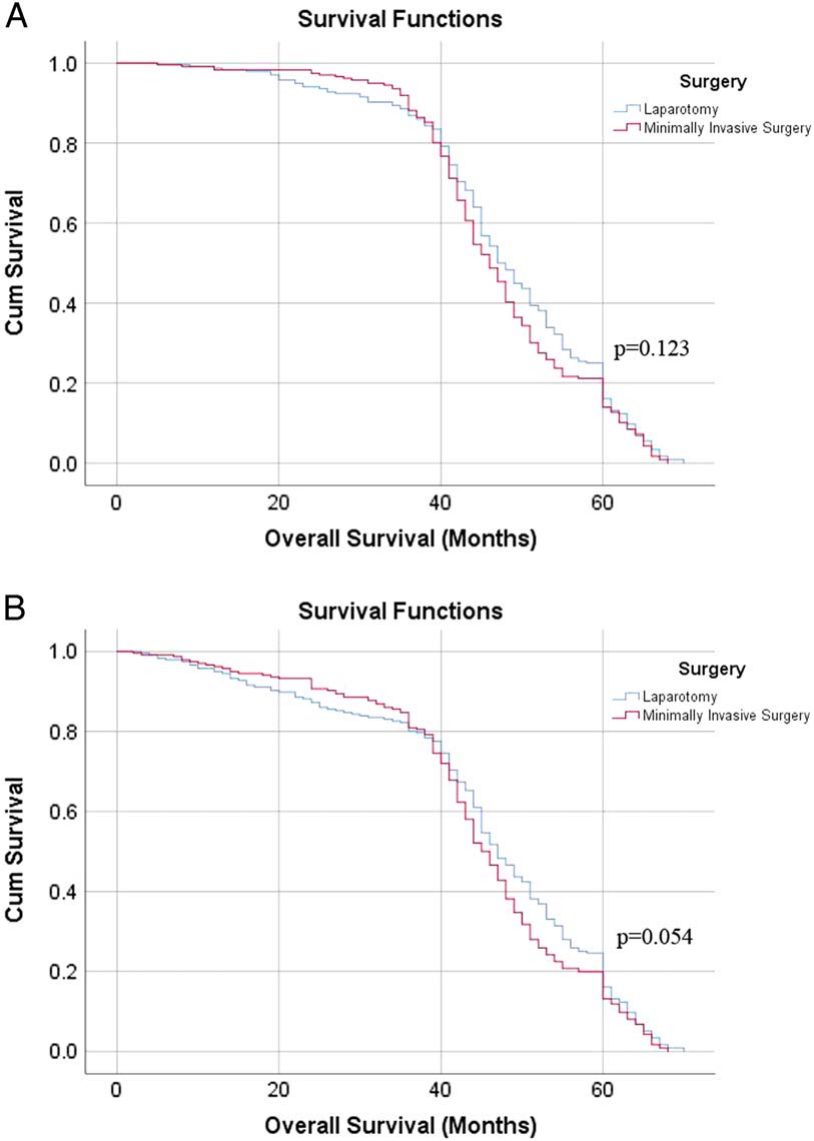
(A) Three-year overall survival curve for patients with colorectal cancer . Of 454 patients, 227 patients were treated by laparotomy, and 227 patients treated by minimally invasive surgery. Insignificant difference was observed in 3-year overall survival, and the median survival time was 45 months versus 47 months (*P*=0.123). (B) Three-year disease-free survival curves for patients with colorectal cancer. There was insignificant difference between the two groups, and the median survival time was 45 months versus 47 months (*P*=0.054).

The survivals of patients are shown in Table 5. The median follow-up period was as follows: MIS group: 44 months, laparotomy group: 50 months. One-year, two-year, three-year and five-year OS and disease-free survival (DFS) did not differ significantly (Table 5). The 3-year survival curve of OS and DFS were shown in Figure 3A, B, the differences in median survival time were insignificant (OS: laparotomy group: 45 months vs. MIS group: 47 months, *P*=0.123; DFS: laparotomy group: 45 months vs. MIS group: 47 months, *P*=0.054). Finally, the recurrences or metastasis were shown in Figure 2B (percentage of total recurrence or metastasis). The most common metastatic site was the liver (21%), followed by the anastomotic site (16%), lung (13%), peritonea (12.5%), lymphatic metastasis (10%), bone (8%), multiple metastasis (11%), and others.

**Table 5:** Long-term survival analysis.

	All (%)	Laparotomy (%)	Minimally invasive surgery (%)	*P*
One-year survival analysis (*n*)	556	278	278	
OS rate	97.6	97.4	97.8	0.584
DFS rate	94.1	94.2	94.1	0.983
Two-year survival analysis (*n*)	544	272	272	
OS rate	92.6	92.2	93.0	0.481
DFS rate	85.6	85.8	85.2	0.676
Three-year survival analysis (*n*)	454	227	227	
OS rate	89.3	88.1	90.6	0.077
DFS rate	83.3	83.2	83.5	0.866
Five-year survival analysis (*n*)	72	36	36	
OS rate	75.0	77.8	72.2	0.285
DFS rate	73.6	75.0	72.2	0.599

*Influence factors for OS and DFS*

Table 6 shows the results of univariate and multivariate analyses of factors affecting OS in patients with CRC who were treated with different surgery options. The age equal or greater than 60 years old (HR 0.533, 95% CI 0.387–0.733; *P*<0.001), lymph node metastasis (HR 0.442, 95% CI 0.323–0.606; *P*<0.001) and CEA greater than 5 ng/ml (HR 0.699, 95% CI 0.517–0.945; *P*=0.020) were independent risk factors for OS in patients with CRC.

**Table 6:** Univariate and multivariate analyses of factors affecting overall survival in patients.

	Univariate		Multivariate	
Variable	HR (95% CI)	*P*	HR (95% CI)	*P*
Sex (Male)	0.800 (0.580–1.104)	0.175		
Age (≥60)	1.888 (1.363–2.617)	<0.001	0.533 (0.387–0.733)	<0.001
BMI	5.048 (0.336–75.804)	0.242		
Tumor location (LCC)	0.860 (0.600–1.233)	0.412		
Differentiation (Well)	0.970 (0.608–1.547)	0.899		
Lymph node metastasis (yes)	2.165 (1.571–2.983)	<0.001	0.442 (0.323–0.606)	<0.001
Tumor diameter longitudinal (cm)	1.048 (0.905–1.212)	0.553		
≥5 cm	1.281 (0.799–2.053)	0.304		
CEA (ng/ml)	0.999 (0.997–1.001)	0.479		
>5 ng/ml	1.414 (1.032–1.939)	0.031	0.699 (0.517–0.945)	0.020
CA125 (ng/ml)	1.002 (0.994–1.009)	0.672		
>40 ng/ml	1.050 (0.468–2.354)	0.906		

Table 7 shows the results of univariate and multivariate analyses of factors affecting DFS in patients with CRC who were treated with different surgery options. Lymph node metastasis (HR 0.453, 95% CI 0.331–0.620; *P*<0.001) and CEA greater than 5 ng/ml (HR 0.686, 95% CI 0.508–0.926; *P*=0.014) were independent risk factors for DFS in patients with CRC.

**Table 7:** Univariate and multivariate analyses of factors affecting disease-free survival in patients.

	Univariate		Multivariate	
Variable	HR (95% CI)	*P*	HR (95% CI)	*P*
Sex (Male)	0.624 (0.431–0.903)	0.012	1.322 (0.996–1.809)	0.081
Age (≥60)	1.055 (0.730–1.526)	0.774		
BMI	1.448 (0.065–32.513)	0.815		
Tumor location (LCC)	1.387 (0.886–2.169)	0.152		
Differentiation (Well)	1.004 (0.648–1.557)	0.984		
Lymph node metastasis (yes)	0.415 (0.279–0.617)	<0.001	0.453 (0.331–0.620)	<0.001
Tumor diameter longitudinal (cm)	1.023 (0.855–1.266)	0.802		
≥5 cm	1.374 (0.762–2.477)	0.291		
CEA (ng/ml)	0.999 (0.996–1.003)	0.725		
>5 ng/ml	0.706 (0.483–1.031)	0.072	0.686 (0.508–0.926)	0.014
CA125 (ng/ml)	0.998 (0.989–1.008)	0.719		
>40 ng/ml	1.139 (0.406–3.193)	0.804		

*Clinical predicition model of OS and DFS*

**Figure 4:**
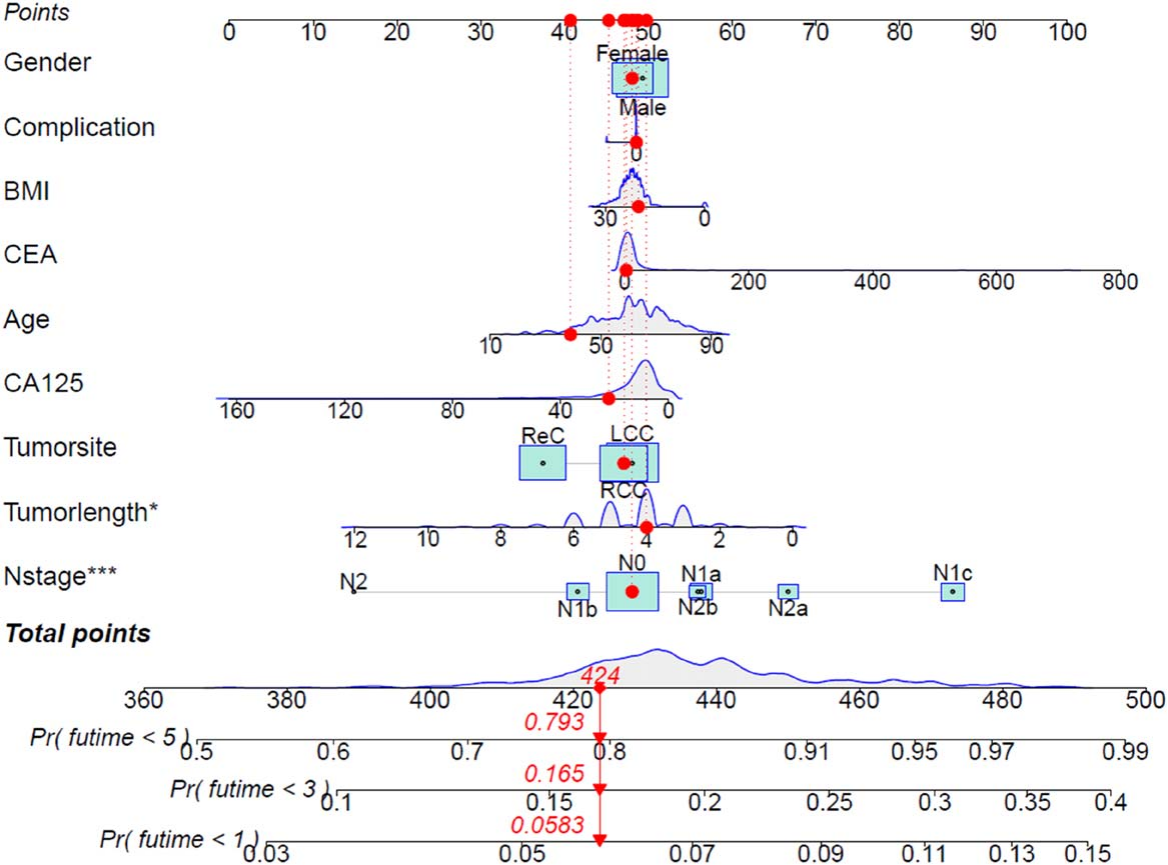
Nomogram of overall survival.

Figure 4 shows the correlation between the sum of scores of sex, age, complications, BMI, CEA, age, CA125, tumor site, tumor length diameter, and N stage and 1-year, 3-year, and 5-year mortality of patients. Among them, tumor length diameter and N stage are significantly correlated with long-term survival of patients.

**Figure 5:**
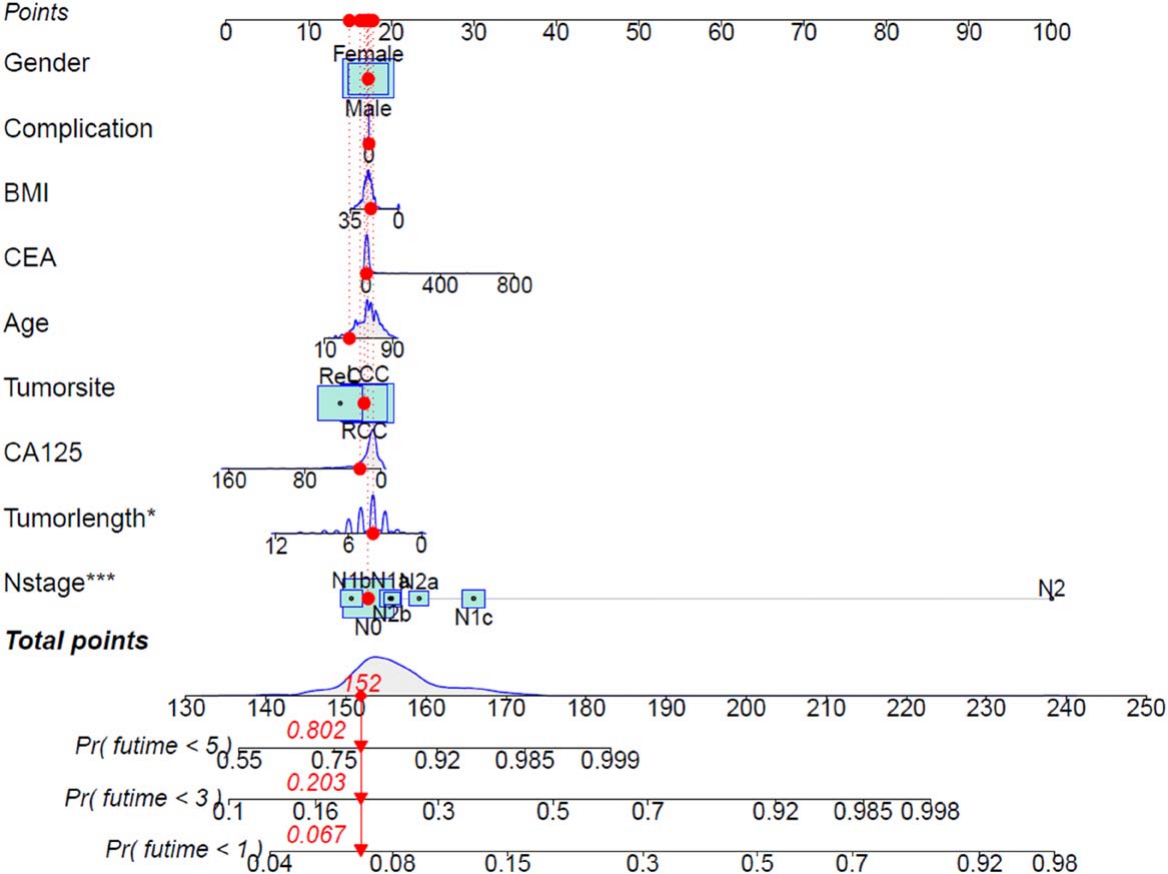
Nomogram of disease-free survival.

Figure 5 shows the correlation between the sum of scores of sex, age, complications, BMI, CEA, age, CA125, tumor site, tumor length, diameter, and N stage and 1-year, 3-year, and 5-year tumor-free survival of patients, among which tumor length diameter and N stage are significantly correlated with the tumor-free survival time of patients.

## Discussion

The results of this study proved that MIS has a better short-term prognosis than laparotomy and comparable long-term survival, with lymph node metastasis rate and CEA levels as independent risk factors for OS and DFS.

In recent years, with the development of MIS and ERAS, MIS (including: common laparoscopy, 3D laparoscopy, 4K laparoscopy, single-port laparoscopy, Da Vinci robotic surgery, and single-port Da Vinci robotic surgery) has been highly used in the surgical treatment of CRC. Besides, with advancements in medical device technology and industry norms, and the continuous learning of medical staff, MIS with its minimal trauma and rapid recovery advantages is increasingly used in the surgical treatment of CRC. We believe that MIS has a clear operative field of vision, allowing for accurate identification of anatomical landmarks, with the added benefits such as reduced intraoperative bleeding, less incisional trauma to patients, faster postoperative recovery, less postoperative complications, and less inflammation, thus is superior when compared with open surgery or laparotomy in the treatment of tumors. Our research came to the same conclusion that the intraoperative blood loss, first exhaust time, first defecation time, postoperative hospital stay time, drain removal time, and incidence of complications in the MIS group are less than in the laparotomy group. However, due to factors such as instrument preparation time, the time of the laparotomy group was longer than that of the MIS group.

In this study, we used PSM to equalize the baseline covariates of patients to reduce selection bias. PSM is a statistical method that mimics randomized clinical trials, effectively reducing confusion bias and increasing credibility^18–20^.

This study showed that lymph node metastasis was an independent risk factor for OS and DFS (*P*<0.001), which is consistent with many studies. Many studies indicate that lymph node metastasis is not only a risk factor for postoperative recurrence and metastasis, but also closely related to its prognosis^21–28^. Therefore, Resch and Langner^29^ indicated that lymph node metastasis had become an important reference for the formulation of postoperative chemoradiotherapy for CRC. Today, we all agree that the standard treatment for resectable CRC is radical excision with systematic dissection of lymph nodes. This is consistent with what we currently know: CRC with lymph node metastases usually signifies a late-stage tumor, and is often used as one of the criteria for evaluating postoperative recurrence and subsequent chemotherapy strategies.

The results of multifactorial studies showed that a high level of CEA is an independent risk factor for nonmetastasis pT4a CRC (OS: *P*=0.020; DFS: *P*=0.014). In recent years, serum CEA level has been considered as an effective prognostic factor for CRC. There were studies suggesting that preoperative CEA expression level was strongly associated with CRC prognosis, and was a reliable basis for treatment as well^30–35^. CEA is a glycoprotein that is present in high levels in gastrointestinal malignancies and it is mainly secreted by solid tumor, which has since been recommended as a reliable tumor marker by the National Comprehensive Cancer Network (NCCN) and the American Society of Clinical Oncology.

Age (60 or older) was considered an independent risk factor for OS in this study (*P*<0.001). Physical function, nutritional status, immunity, and overall health of patients after the age of 60 are generally weaker than those before. This may be because older patients, with worse immunity and resistance, cannot handle the extensive damages caused by the operation and subsequent postoperative comprehensive treatment, leading to reduced life expectancy. Some might even delay treatment due to poor economic conditions and poor compliance. In addition, the older group of patients has more comorbidities (such as high blood pressure, diabetes, heart disease, renal insufficiency, cirrhosis, COPD, coronary heart disease, cerebral infarction, and so on), which may increase the incidence of postoperative complications and even lead to a worse prognosis, which may affect the way the principal operator chooses the operation, resulting in bias. However, since this study is a retrospective analysis and cannot be randomized.

With the development of minimally invasive techniques and ERAS, these minimally invasive techniques have become the mainstay treatment for CRC^36–39^. By definition, nonmetastasis pT4a CRC has not invaded other tissues or organs, hence does not involve multiorgan resection, MIS is commonly preferred. However, MIS for the treatment of nonmetastasis pT4a CRC remains controversial due to the lack of strong evidence-based studies and the uncertainty to achieve R0 resection^13–16,40,41^. In this study, the intraoperative blood loss (*P*<0.001), first exhaust time (*P*<0.001), first defecation time (*P*=0.006), drainage tube removal time (*P*<0.001), postoperative hospital stay time (*P*<0.001), and complication rate (*P*=0.010) in the MIS group were lower than those in the laparotomy group. Moreover, the time of laparotomy was shorter than that of MIS (*P*<0.001), and the number of lymph nodes removed was not statistically significant (*P*=0.527). These results were partly consistent with previous studies^13–16,42–44^. These studies have shown that MIS can provide a clear surgical field for the accurate identification of anatomical landmarks, resulting in less intraoperative blood loss, less incisional trauma, faster postoperative recovery, less postoperative complications, and similar or better clinical results when compared to laparotomy. Many studies agreed that there were no significant differences in the long-term survival outcomes^13–16,40,45–48^. The conversion rate in our study was 3.5%, which did not exceed what had been reported in other studies^49–53^.

Complications are associated with short-term outcomes and long-term survival^54–58^. Its occurrence will cause various pathophysiological and psychological reactions in patients, which will affect the surgical efficacy and survival, even potentially induce other serious diseases if left unchecked. Our findings suggested that common complications included anastomotic fistula, intra-abdominal infection, incision infection, and so on.

Tumor recurrence or metastasis occurs when original tumor cells proliferate in local, regional, or distant tissues, perhaps through the lymph nodes, circulation, or abdominal spread^59–61^. Recurrence of CRC is defined as local, regional, or distant metastasis after a disease-free period^62–64^. The most commonly reported local recurrence was lymph node metastasis, and the most commonly reported distant metastases were the liver, the lungs, and so on. Similar results were found in our study, with the most common postoperative recurrence or metastasis found in the liver, followed by abdominal metastasis and lung metastasis.

With technological advancements, MIS is now capable of better surgical exposure, resulting to a lesser surgical trauma and a faster recovery. For example, in robotic surgery, a well-trained surgeon can operate all four arms by himself, reducing the inaptitude brought on by an inexperienced assistant, hence ensuring a comfortable operating environment for the surgeon and enabling a smooth and eventless operation.

This study has some limitations, including the classification of both laparoscopic and robotic surgery as MIS and not being analyzed separately. All patients are from a single center, but they are in the different treatment groups with surgeons of different skill levels. It is not an randomized clinical trials, and we could not measure the experience of individual surgeons on the MIS.

## Conclusion

The study demonstrated favorable short-term and long-term outcomes of MIS for nonmetastatic pT4a CRC. In summary, MIS is considered to be a valid option.

## Ethical approval

This study was approved by the Medical Ethics Committee of the Seventh Affiliated Hospital of Sun Yat-sen University (No. KY-2023-053-01).

## Consent

Individual consent for this retrospective analysis was waived.

## Sources of funding

Guangdong Provincial Key Laboratory of Digestive Cancer Research (No. 2021B1212040006), Sanming Project of Medicine in Shenzhen (No. SZSM201911010), Shenzhen Key Medical Discipline Construction Fund (No. SZXK016), Guangdong–Hong Kong–Macau University Joint Laboratory of Digestive Cancer Research (2021LSYS003), and Shenzhen Fundamental Research Program (JCYJ20190809142807444).

## Author contribution

H.-L.G., J.-Y.C., Y.-Z.T., and W.-H.W.: contributed equally to this study, and both conceptualized and designed the study, analyzed and interpreted the data, drafted the manuscript, and critically revised the manuscript for important intellectual content; Q.-L.Z., Q.-L.J., and M.-Z.L.: participated in data acquisition and statistical analysis; Y.-L.H.: supervised the whole study and monitored the standard surgical operations. All the authors took part in the surgical treatment of colorectal cancer.

## Conflicts of interest disclosure

The authors have no conflict of interest related to the manuscript.

## Research registration unique identifying number (UIN)

No. ChiCTR2300073447.

## Guarantor

Wen-Hui Wu, MD, Digestive Diseases Center, The Seventh Affiliated Hospital of Sun Yat-sen University, Shenzhen 517018, Guangdong Province, People’s Republic of China. E-mail: doctorwusysu@126.com.

## Data availability statement

The original anonymous dataset is available on request from the corresponding author at doctorwusysu@126.com.

## Provenance and peer review

Yes.
